# International survey of diagnostic services for children with Fetal Alcohol Spectrum Disorders

**DOI:** 10.1186/1471-2431-8-12

**Published:** 2008-04-15

**Authors:** Elizabeth Peadon, Emily Fremantle, Carol Bower, Elizabeth J Elliott

**Affiliations:** 1 Australian Paediatric Surveillance Unit, The Children's Hospital at Westmead, Locked Bag 4001, 2145 Westmead, Australia; 2 Discipline of Paediatrics and Child Health, University of Sydney, Australia; 3 Telethon Institute for Child Health Research, Centre for Child Health Research, University of Western Australia, Perth, Australia

## Abstract

**Background:**

Early diagnosis and intervention for children with Fetal Alcohol Spectrum Disorder (FASD) reduces the risk of developing a range of secondary social, emotional and behavioural problems and provides an opportunity for prevention of further alcohol exposed pregnancies. The objective of this study was to describe specialist clinical service provision for the diagnosis and assessment of children exposed to alcohol in pregnancy.

**Methods:**

Fetal Alcohol Spectrum Disorder (FASD) diagnostic clinics were identified through literature and internet searches. Clinics were sent a questionnaire asking for information on the clinic population, clinic staff, assessment process and other services provided.

**Results:**

Questionnaires were completed for 34 clinics: 29 were in North America, 2 in Africa, 2 in Europe and 1 in South America. No clinics were identified in Asia or Australasia. There was a variety of funding sources, services offered, clinic populations, staff and methods of assessment. Thirty-three clinics had a multi-disciplinary team. In 32 clinics, at least one member of the team had specialist training in assessment of FASD. Neurobehavioural assessment was completed in 32 clinics. Eleven clinics used more than one set of diagnostic criteria or an adaptation of published criteria.

**Conclusion:**

Diagnostic services are concentrated in North America. Most responding clinics are using a multidisciplinary approach with neurobehavioural assessment as recommended in published guidelines. Agreement on diagnostic criteria would enable comparison of clinical and research data, and enhance FASD research particularly for intervention trials.

## Background

The effects on the fetus of alcohol exposure in pregnancy have been well described but barriers to diagnosis remain. Fetal Alcohol Spectrum Disorder (FASD) includes Fetal Alcohol Syndrome (FAS), alcohol-related neurodevelopmental disorder (ARND) and alcohol-related birth defects (ARBD), and is said to affect 1% of all live births in the United States of America (USA) [1]. Prevalence rates of Fetal Alcohol Syndrome (FAS) are reported to be between 0.06 [2] and 0.68 [3] per 1,000 live births in Australia and 0.5 and 2 per 1,000 live births in USA [1]. Higher prevalence rates have been reported in populations in Italy (3.7 to 7.4 per 1,000 children) [4], South Africa (68.0 to 89.2 per 1,000 children in Cape Province) [5] and some indigenous populations (Plains Indian: 9.0 per 1,000 live births [1]; Indigenous Australians: 2.76 [6] to 4.7 [3] per 1,000 live births).

Fetal Alcohol Syndrome costs the USA US$3.6 billion per year and the total lifetime cost for an individual with FAS is estimated at US$2.9 million [7]. The costs of FAS include health care, residential and support services, developmental disability services, special education, social services, adult vocational services and productivity losses [7]. Much of this cost is attributable to the secondary disabilities of FAS including disrupted education, contact with the law, mental health problems, alcohol and drug misuse, inappropriate sexual behaviour and inability to obtain and maintain employment and independent living [8-10]. Early diagnosis of FASD may reduce the odds of experiencing these adverse outcomes by two- to four-fold [10].

Guidelines regarding the assessment of children with suspected FASD have been published in the USA [11-14] and Canada [15]. Recommended standards for assessment include multidisciplinary teams who have specific training in assessing children exposed to alcohol *in utero*, can assess the child's and family's strengths and needs, and make appropriate referrals for further management. The aim of our study was to conduct an international survey to describe specialist dedicated clinical service provision for the diagnosis and assessment of children with FASD; to establish which countries have specialised services and describe the models of service used; and compare clinical practice in the services with the published recommendations for assessment of children exposed to alcohol *in utero*.

## Methods

Clinics which provided a dedicated specialist service for the assessment of children exposed to alcohol *in utero *were included in this study. Diagnostic and assessment clinics were identified by searching four literature databases: MEDLINE (1950 to 2006), CINAHL (1982 to 2006), EMBASE (1980 to 2006) and PsychINFO (1985 to 2006). The term "Fetal Alcohol Syndrome" was combined with "Health Services" or "Diagnosis". The internet was searched for diagnostic clinics and clinic evaluation reports using the Google search engine. In countries in which no clinic was identified in the initial search, researchers identified through publications about FASD or on the internet and organisations involved with people with a FASD were contacted for information regarding diagnostic services in their country.

A questionnaire was designed to collect data from identified clinics on: the clinic population (number of children seen, age range, referral process); clinic staff (number, professional group, specific training in diagnosis of FASD, role in clinic); assessment process (number and length of visits, assessment tools and diagnostic criteria used); and other clinic activity (screening, management and research). Contact details of other clinics known to respondents were also sought. The questionnaire was a structured, three page, self- administered instrument which could be completed electronically or by hand. The questionnaire was piloted as a structured telephone interview prior to sending to all clinics. All contact with clinics was via email. The questionnaire is available on request from the authors.

**Figure 1:**
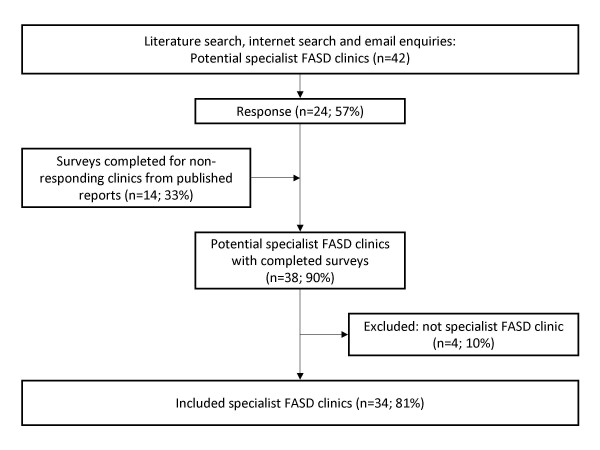
Survey process and response.

Figure 1 shows the survey process. The questionnaire was sent to identified clinics between June and December 2006 inclusive. When a network of clinics was identified, the principal clinics in that network were contacted. Clinics that did not respond were contacted up to 3 times and if there was still no response the questionnaire was completed using published clinic evaluation reports when available [16-19]. Clinics that were not specific FASD diagnostic services (e.g. general dysmorphology or genetic or child development clinics) were not eligible for inclusion in this survey.

## Results

Contact was made with clinics identified in the literature search, research clinics linked to these, and potential clinics identified after general enquiry. We had responses to our enquiries stating that there was no identified specialist FASD diagnostic service from Europe (Denmark, Finland, France, The Netherlands, Norway, Russia, Spain, Sweden, Switzerland, Ukraine); Asia (Japan) and Australasia (Australia, New Zealand). No response was received from Brazil and Germany. We received 20 completed questionnaires from eligible clinics and completed questionnaires for 14 further clinics using published evaluation reports identified in the search (Figure 1). Of the 34 clinics, 24 clinics were from the USA, five from Canada and five from outside North America (United Kingdom, Italy, Chile, South Africa) (Table 1). The completed questionnaires included one group of three linked research clinics in the USA, South Africa and Italy, which were included as separate clinics. Two additional clinics in the USA had external research clinics. However, there was insufficient information for inclusion of these sites as separate clinics. Aggregate data was available on one state clinic network in the USA but individual clinic data was not available for the eleven teams within the network.

**Table 1:** Clinic Characteristics

**Clinic Site**	**Funding Source**	**Services Offered**	**Training for Professionals**	**Training for Parents**	**Referral Criteria**	**Ages Seen**	**New referrals seen per year**	**% of Children seen with FAS/FASD**
**Canada**								
Province A^13^	State, fee for service	Diagnosis, short term management	Yes	NR	None	All ages	60	NR
Province B^14^	State, research, community	Diagnosis, short term management	No	No	Children with verified prenatal exposure to alcohol selected from the waiting list for the Psychology Clinic	6 to 16 yrs	15 in pilot period	NR
Province C	Federal, charitable	Screening, diagnosis, management	NR	Yes	Children of drug treatment programme only	0 to 6 yrs	50 to 75	NR
Province C^16^	Federal funding now ceased; clinic not operational	Diagnosis, short term management	Yes	NR	None	0 to 12 yrs	49	3/77
Province C	State	Screening, diagnosis, management	Yes	Yes	Prenatal alcohol exposure & evidence of CNS (behaviour)	<18 yrs	40- to 50	Unknown, most would have ARND

**USA**								
State A^a^	Federal	Diagnosis, short term management	Yes	No	Pre-natal alcohol exposure	Usually <18 years	150 across network	2.6/70
State B	Self-pay, insurance	Diagnosis	Yes	Yes	None	0 to 12 yrs	35	10/90
State B^b^	Insurance, research grants	Screening, diagnosis, management	Yes	No	None	All ages	100	10/10
State B	Fee for service	Diagnosis	Yes	No	None	0 to 21 yrs	50	25/25
State B	State	Screening, diagnosis	Yes	No	Prenatal alcohol exposure and contacted telephone information service	All ages	NR	1/5
State C	State & federal funding, fee for service, insurance	Screening, diagnosis, management	Yes	Yes	Prenatal exposure to alcohol or other drugs	0 to 21 yrs	200 to 250	45/30
State D^b^	Research grants	Screening, diagnosis	No	No	Children of heavy drinkers	NR	NR	NR
State E	Fee for service, charitable foundation	Diagnosis	Yes	No	FASD highly suspected, but may not be confirmed.	0 to 21 yrs	80	5/87
State F	State & federal	Screening, diagnosis	Yes	Yes	None	0 to 20 yrs	100	20/30
State G^c^	Federal	Screening, diagnosis	Yes	NR	Developmental delay, growth parameters < 25th centile, known prenatal alcohol exposure, or previous diagnosis of FAS/FASD	0 to 18 yrs	300	0.7/3.7
State H	State & federal funding, fee for service	Screening, diagnosis, management	Yes	Yes	None	All ages	300 to 400	10/15
State I	Sliding fee scale, adoption subsidy, children services, contributions	Screening, diagnosis, management	Yes	Yes	Prenatal Alcohol exposure suspected then referrals screened for suitability	3 to 18 yrs	20	20/80
State J	State	Screening, diagnosis	Yes	Yes	Any prenatal alcohol exposure	All ages	60 families	9/vast majority
State K	Contract, federal, state, fee for service	Screening, diagnosis, short term management	Yes	Yes	Prenatal alcohol exposure, developmental &/or behavioural concerns	3 to 16 yrs	24	5/95

**Chile**								
Clinic A	Research grants (US NIH)	Screening, diagnosis, management	Yes	Yes	Protocol (unspecified)	0 to 5 yrs	20	8/25

**South Africa**								
Clinic A	Research grants (US and local)	Screening, diagnosis, management	Yes	Yes	Birth records from local hospitals	9 to 24 months & 7 to 12 yrs	Recruited, not referred	Multiple sites, range: 1.9 to 10.3/2.5 to 12.2
Clinic B^c^	Federal (US Research)	Screening, diagnosis, management	Yes	NR	Developmental delay, growth parameters < 25th centile, known prenatal alcohol exposure, or previous diagnosis of FAS/FASD	0 to 18 yrs	1600	7.4/NR

**Italy**								
Clinic A^c^	Federal (US Research)	Screening, diagnosis	Yes	NR	Developmental delay, growth parameters <25th centile, known prenatal alcohol exposure, or previous diagnosis of FAS/FASD	0 to 18 yrs	543	4/NR

**United Kingdom**								
Clinic A	National Health Service	Diagnosis	Yes	No	None	14 to 18 yrs	12 (6 months only)	0/100

NR, no response to the question.

^a ^Aggregate data for network of 11 clinics.

^b ^These clinics also have research focussed satellite clinics, however insufficient separate information was given for separate inclusion.

^c ^Clinics are a linked research network.

Clinics had a variety of funding sources, services offered, clinic populations, staff and methods of assessment (Table 1). Funding came from many sources, including charitable and community sources in four cases. One clinic had closed because of lack of funding and seven were funded by research grants. Four of the five clinics outside North America were funded partially or wholly by research grants from the USA. Only two clinics, both in the USA, relied wholly on patient fees (self-pay or insurance) and did not receive any state, federal, research or charitable contributions.

All 34 clinics offered a diagnostic service. Sixteen were also involved in screening for at risk children; 15 offered short term management; and nine offered ongoing management (Table 1). Thirty-one clinics offered training to external health professionals and 11 provided training for parents. Twelve clinics provided outreach services, 10 provided case conferencing, six provided home visits, and one provided a telemedicine service.

Referral criteria varied between clinics (Table 1). Eight of the 34 clinics had no specific referral criteria. Of the clinics with referral criteria, some required only a history of pre-natal alcohol exposure, whereas others had more specific criteria mirroring the diagnostic features of FAS (i.e. prenatal alcohol exposure, central nervous system disorder and growth deficiency). Most clinics (n = 27) accepted referrals from multiple sources. Primary care practitioners were the most common referral source (n = 27), followed by specialist paediatricians (n = 24). Other common referral sources included self or family referral (n = 23), child protection services (n = 23), mental health services (n = 22), schools (n = 21), legal services (n = 21), family support groups (n = 11), drug and alcohol services (n = 10), other health professionals (n = 10) and geneticists (n = 8). Five clinics with a research focus did not accept referrals but specifically recruited children exposed to alcohol *in utero *for assessment.

There were differences between the patient populations of clinics (Table 1). The number of new patients seen in clinics each year ranged from 20 to 1600. The rate of diagnosis of FAS ranged from 0.7% to 45% (median 7.4%). The rate of diagnosis of other FASD ranged from 2.5% to 100% (median 25%). Twelve of the 17 clinics who provided estimates of both FAS and other FASD rates in their clinic population stated a higher rate of other FASD than FAS diagnosis. An estimate of the ethnic composition of their clinic population was provided by 18 clinics: 10 reported seeing a majority of Caucasian children; three clinics reported that children seen were most commonly of indigenous origin. Twenty-four clinics reported that the majority of children lived in alternate care (away from their biological parents). In 20 of these clinics, 75% or more of the children seen were in alternate care.

Thirty-three of the 34 clinics were staffed by a multidisciplinary team; however the composition of teams varied (Table 2). All 33 multidisciplinary teams had at least one medical and one psychology professional. The one clinic that was not run by a multidisciplinary team was staffed by a dysmorphologist. At least one member of staff had undergone specialist training for FASD in 32 clinics. In 22 clinics, all members of staff had undergone specialist training.

**Table 2:** FASD Diagnostic Clinic Team Composition (n = 34)

**Type of Staff**	**Number of clinics**
Number of clinics with any Psychology Professional	33
Psychologist	30
Neuropsychologist	10
Psychometrists	1
Number of clinics with any Medical Professional	34
General Paediatrician	21
Geneticist/Dysmorphologist	12
Developmental Paediatrician	8
Nurses	4
Child Psychiatrist	4
Physician	1
Paediatrician/Toxicologist	1
Endocrinologist	1
Ophthalmologist	1
Number of clinics with any Allied Health Professional	23
Occupational Therapist	23
Speech Therapist	23
Physiotherapist	14
Dietician	1
Audiologist	1
Number of clinics with any Family Support Professional	29
Social Worker	22
Mental Health Worker	14
Family/Follow up Support Worker (non-diagnostic)	13
Case Manager	9
Cultural Worker	5
Family Advocate	4
Drug and Alcohol Worker	2
Child Protection Worker	1
Genetic counsellor	1
Child Development Counsellors	1
Home based parent-child therapists	1
Number of clinics with any Education Professional	8
Licensed Educational Diagnostician	3
Teacher	3
Education Specialist	2
Number of clinics with any specified Administrative Support:	7
Clinic/Team Coordinator	7
General administrative support	2
Number of clinics with any additional Research Support Workers:	5
Maternal Interviewer	3
Research Assistant	1
Residents/post doctoral fellows/interns	1

For thirty clinics, information was available on their clinical assessment process. Four clinics did not request any information prior to the visit for assessment. Thirteen of those that required prior information requested medical assessment reports; 12 requested childcare, preschool or school reports; 11 requested developmental or psychometric assessments; nine requested Child Protection Service or Foster Service records; and four clinics requested birth records. The number of visits required for assessment and diagnosis ranged from one to three with a median of one visit. Two clinics responded that the number varied from child to child. The duration of visits was between 0.5 and 6 hours, with a median of 3.25 hours (16 of 23 responses gave a specific numerical duration for each visit; others were either variable or age-specific).

The primary caregiver(s) and the child attended the assessment in all 33 of the clinics for which we had information. In three clinics, siblings also attended the assessment and in one other clinic, siblings were invited to attend if appropriate. Six clinics specified that case managers or social service workers or child protection workers attend the clinic. In fifteen clinics, all members of the multidisciplinary team took part in the diagnostic process. In two clinics, the geneticist/dysmorphologist made the diagnosis alone, and in all other clinics more than one member of the team took part in the diagnostic assessment process.

Clinics had different approaches to the assessment process. Twenty-three reported that they routinely carried out a physical assessment of the child. Twenty-five clinics took facial photographs and seventeen clinics used facial analysis software. Other routine assessments included audiology (n = 7), genetic testing (n = 7), vision assessment (n = 6) and neuro-imaging (n = 5). Thirty-two clinics carried out some neurobehavioural assessment. One clinic did not respond to this question, and one did not carry out any neurobehavioural assessment. Neurobehavioural assessments included: behavioural assessment (n = 28); motor or visual-motor or perception tests (n = 28); sensory function (n = 22); cognitive or developmental testing (n = 19); neuropsychometric tests (n = 17); adaptive behaviour or social skills or social communication (n = 17); communication assessment (n = 13); educational or academic assessment (n = 12); and neurological examination (n = 12).

Information was sought on the diagnostic criteria clinics used. Of the 23 clinics using one set of diagnostic criteria, fourteen were using the Washington 4-digit Diagnostic code (2004) [11], eight were using Hoyme et al's 2005 revision of the 1996 Institute of Medicine criteria [13], one was using the 1996 Institute of Medicine's criteria [14] and none were using the Center for Disease Control's 2004 guidelines [12]. Eleven of the 34 clinics used more than one of the published criteria or an adaptation of published criteria. Two of the clinics using the Washington 4-digit Diagnostic code as their only diagnostic criteria had made adaptations to it. Nine clinics were using a combination of criteria: Washington 4-digit Diagnostic code, Center for Disease Control's 2004 guidelines and 1996 Institute of Medicine (n = 2); diagnostic blend of Hoyme et al, Canadian guidelines, Washington 4-digit Diagnostic code and 1996 Institute of Medicine (n = 1); Hoyme et al, Washington 4-digit Diagnostic code and 1996 Institute of Medicine (n = 1); Canadian guidelines and Washington 4-digit Diagnostic code (n = 1); Washington 4-digit Diagnostic code and 1996 Institute of Medicine (n = 1); Hoyme et al and 1996 Institute of Medicine (n = 1); Center for Disease Control's 2004 guidelines and 1996 Institute of Medicine (n = 1); 1996 Institute of Medicine and other criteria (n = 1).

Of the 34 clinics included in the survey, fifteen offered short-term management and nine offered longer-term management. Of the 24 clinics offering management to their patients following diagnosis, a variety of interventions were used. The most common intervention was provision of family support services (n = 10). Other management offered included counselling or behaviour management (n = 6), speech therapy (n = 5), case management (n = 4), physiotherapy (n = 4), occupational therapy (n = 4), child and adolescent mental health services (n = 4), and drug and alcohol (n = 3). For clinics that did not offer management (n = 10), referrals for follow up care and management were made to local services (n = 2), state services (n = 1), to the school district (n = 2) or back to the primary care practitioner (n = 1).

## Conclusion

Despite evidence that early diagnosis and intervention may be beneficial to children with FASD [10], specialist diagnostic clinics for FASD are largely limited to North America. All but one of the diagnostic clinics found outside North America were research based and funded either partially or wholly by research grants from the USA.

In regions which lacked specialist clinics, particularly Europe, there was interest and work towards establishing diagnostic and assessment services.

Making a diagnosis of the conditions resulting from alcohol exposure *in utero *can be difficult for an untrained health professional. Many of the features of these conditions can be seen in a number of other genetic and malformation syndromes, and it can often be hard to ascertain pre-natal exposure to alcohol use [12]. The diagnosis of ARND can be particularly challenging due to the absence of physical signs. Thirty-two of the clinics stated that at least some members of the diagnostic team had undergone specialist diagnostic training.

Surveys of child health professionals demonstrate their lack of knowledge about the diagnostic features of FAS [20-23]. American studies indicate good general knowledge about FAS but hesitancy to make a diagnosis [21,22]. Australian surveys demonstrate a lesser knowledge of the diagnostic features and a similar hesitancy to make a diagnosis [20,23]. These surveys also identify that health professionals feel poorly equipped to manage a patient with FAS [20-23]. Health professionals would like more resources including information on FAS, referral services and a register of health professionals with expertise in diagnosing FAS [20].

The diagnosis of a FASD is complicated by the debate about the most appropriate diagnostic criteria, with multiple guidelines published since 1996 [11-15,24]. This complexity was reflected in this survey. Notably, several clinics used more than one set of diagnostic criteria or their own adaptations of published criteria. The Center for Disease Control's 2004 guidelines only define diagnostic criteria for FAS [12]. Services which use these criteria would need to use another set of diagnostic criteria for other FASD diagnoses. Only two of the clinics using a combination of criteria, were using the same combination of criteria. This lack of agreement in diagnosis reduces the potential for comparison of data about FASD across clinics and countries. It also highlights the potential for confusion for health professionals around the diagnosis of FASD.

Of the 34 clinics, 33 were staffed by a multi-disciplinary team. All the clinics with a multi-disciplinary team had medical and psychology professionals but not all had allied health professionals, family support services and education professionals recommended in the literature [12,15,25]. The clinics are generally using a multidisciplinary approach with some form of neurobehavioural assessment as recommended in published guidelines [11-13,15,25].

There was a variety of ways of delivering the service to the community. Some clinics were situated within the community for which it provided diagnosis and assessment. Some clinics were based in large centres and provided outreach clinics to smaller centres, or, in one case, a telemedicine service. This survey was not designed to capture diagnostic and assessment services provided within general genetic, paediatric or child development clinics. However, we know that these clinics do see children exposed to alcohol *in utero*, especially in areas without specialised clinics. Training needs to be accessible to all health professionals who are in the position to identify children who need assessment, as well as the health professionals who are providing diagnosis and assessment.

Our study has limitations. A range of countries was represented in the study, but not every country where FASD has been reported. Although we asked identified clinics, researchers and FASD organisations for contacts details of relevant services, our search strategy was biased towards services which had the resources to develop a website or publish research. This was more likely to exclude small clinics and clinics in developing countries. All communication was made in English and by email which excluded health professionals who do not read English or do not have email and internet access. The response rate was disappointing and only two respondents suggested other clinics to contact, leading to significant gaps in the data. There was a poor response rate from Canadian clinics where there is a large amount of clinical activity. Data for fourteen clinics was obtained only from published reports i.e. they did not respond to the questionnaire. All these factors affect the representativeness of the sample. The question regarding the rate of FASD diagnoses was intended to include children with a FASD diagnosis other than FAS. However, the wording of the question was ambiguous and thus some FASD rates may include FAS while others do not. Interestingly, higher rates of all FASD diagnoses in a clinic did not correspond with stricter referral criteria.

This study provides the first overview of the international provision of specialist services for diagnosis and assessment of children exposed to alcohol *in utero*. Specialist diagnostic and assessment services are concentrated in North America, and clinics outside North America are mostly dependent on research funding from the USA. Where specialist services do exist, there is a considerable variation in diagnostic practice but there is strong support for a multidisciplinary approach by trained professionals. The variation in use and application of diagnostic criteria is a key issue which needs to be further addressed by clinicians and researchers in this field to promote consistency in diagnosis, and allow international comparison of clinical and research data. This is particularly relevant for epidemiological studies and evaluation of specific interventions for children with a FASD.

There is a range of possible models for the provision of FASD assessment services for children including assessment by: individual health professionals; general child assessment services such as child development or genetic services; or specialist FASD assessment services. Within each of the models, there are several options, for instance: type of health professional(s), assessment process and diagnostic criteria. Planners need to assess the possible models or mixture of models that would suit their context and identify strategies to ensure funding, sufficient numbers of appropriately trained professionals and access for dispersed populations. If a specialist assessment service model is chosen, planners should consider using a multidisciplinary team and provision of specialist training in FASD diagnosis and assessment for team members. Whichever service model is used, health professionals should be educated and supported to identify and provide appropriate services to children exposed to alcohol *in utero*. Finally, implementing consistent diagnostic criteria will enable collaborative research and meaningful comparison of clinic outcomes.

## Competing interests

The author(s) declare that they have no competing interests.

## Authors' contributions

EP designed the study, created the survey, collected and analysed data and wrote the first draft of the text. EF collected and analysed data and wrote the first draft of the text. CB and EJE conceived of and designed the study, assisted in the creation of the survey, and edited and revised the text. All authors read and approved the final manuscript.

## Pre-publication history

The pre-publication history for this paper can be accessed here:


